# Non–antigen-contacting region of an asymmetric bispecific antibody to factors IXa/X significantly affects factor VIII-mimetic activity

**DOI:** 10.4161/19420862.2015.989028

**Published:** 2014-12-18

**Authors:** Zenjiro Sampei, Tomoyuki Igawa, Tetsuhiro Soeda, Miho Funaki, Kazutaka Yoshihashi, Takehisa Kitazawa, Atsushi Muto, Tetsuo Kojima, Satoshi Nakamura, Kunihiro Hattori

**Affiliations:** 1 Research Division; Chugai Pharmaceutical Co., Ltd; Tokyo, Japan; 2 Department of Bioengineering; Tokyo Institute of Technology; Yokohama, Japan

**Keywords:** antibody engineering, bispecific antibody, constant region, disulfide bond, elbow angle, Fc glycosylation, flexibility, hemophilia A, hinge, IgG subclass

## Abstract

While antibody engineering improves the properties of therapeutic antibodies, optimization of regions that do not contact antigens has been mainly focused on modifying the effector functions and pharmacokinetics of antibodies. We recently reported an asymmetric anti-FIXa/FX bispecific IgG_4_ antibody, ACE910, which mimics the cofactor function of FVIII by placing the two factors into spatial proximity for the treatment of hemophilia A. During the optimization process, we found that the activity was significantly affected by IgG subclass and by modifications to the inter-chain disulfide bonds, upper hinge region, elbow hinge region, and Fc glycan, even though these regions were unlikely to come into direct contact with the antigens. Of these non–antigen-contacting regions, the tertiary structure determined by the inter-chain disulfide bonds was found to strongly affect the FVIII-mimetic activity. Interestingly, IgG_4_-like disulfide bonds between Cys131 in the heavy chain and Cys114 in the light chain, and disulfide bonds between the two heavy chains at the hinge region were indispensable for the high FVIII-mimetic activity. Moreover, proline mutations in the upper hinge region and removal of the Fc glycan enhanced the FVIII-mimetic activity, suggesting that flexibility of the upper hinge region and the Fc portion structure are important for the FVIII-mimetic activity. This study suggests that these non–antigen-contacting regions can be engineered to improve the biological activity of IgG antibodies with functions similar to ACE910, such as placing two antigens into spatial proximity, retargeting effector cells to target cells, or co-ligating two identical or different antigens on the same cell.

## Abbreviations

FVIII: coagulation factor VIII
FIX: coagulation factor IX
FIXa: activated coagulation factor IX
FX: coagulation factor X
FXa: activated coagulation factor X
FAE: Fab-arm exchange

## Introduction

Various drug-related properties of therapeutic IgG antibodies, such as their antigen-binding properties, pharmacokinetics, pharmaceutical properties, immunogenicity, and effector functions, can be improved by antibody engineering and optimization technologies. These technologies can be divided into two categories: variable region engineering and constant region engineering. Variable region engineering provides higher or appropriate levels of binding affinity to targets, a longer plasma half-life, improved pharmaceutical properties, and reduced immunogenicity.^1^ Constant region engineering can also provide better efficacy or safety and a longer plasma half-life by selecting the appropriate subclass of IgG and modifying the affinity to each Fc receptor.^2,3^ Engineering the regions that do not have contact with antigens has been mainly concerned with modifying the effector functions, such as antibody-dependent cell-mediated cytotoxicity (ADCC) and complement-dependent cytotoxicity (CDC), or with altering the plasma half-life of IgG antibodies. In fact, when the tertiary structure of whole IgG is crucial to its biological activity, engineering the constant region (or non–antigen-contacting region) by modifying its tertiary structure of IgG (angle and distance between the two Fv domains, flexibility, etc.), could play an important role in its biological activity. However, a limited number of works have been reported in this area.^4,5^

We recently reported that a novel asymmetric bispecific IgG antibody, ACE910, which recognizes activated coagulation factor IX (FIXa) and coagulation factor X (FX) with separate arms, is able to mimic the cofactor function of coagulation factor VIII (FVIII) and demonstrates a hemostatic effect in cynomolgus monkeys.^6-9^ ACE910 is currently being tested in a clinical study as a drug candidate for the treatment of hemophilia A. Similarly to the cofactor function of FVIII,^10^ ACE910 supports FIXa to activate FX by interacting with FIXa and FX with adequate affinity and by placing these two factors into spatially appropriate positions. Asymmetric bispecific IgG antibodies that mimic the cofactor function of FVIII were screened from a large panel of bispecific combinations of anti-FIXa and anti-FX monoclonal antibodies.^7^ The human IgG_4_ variant was selected as the constant region of this molecule because, when compared to other human IgG subclasses, IgG_4_ has fewer effector functions,^2^ which should be avoided considering the mode of action of this bispecific antibody. These bispecific antibodies consist of two different heavy chains and two identical common light chains. The anti-FIXa heavy chain (hereinafter, Q chain) and the common light chain (hereinafter, L chain) make up the FIXa binding site. The anti-FX heavy chain (hereinafter, J chain) and the L chain compose the FX binding site. Mutations are introduced into the C_H_3 region to promote heterodimerization of the Q and J chains.^7^

The cofactor activity of activated coagulation factor VIII (FVIIIa) is to promote FIXa-catalyzed FX activation. We termed this promoting activity “FVIII-mimetic activity,” and during the optimization process of the lead antibody of ACE910, we expended great effort to improve the FVIII-mimetic activity to a level sufficient for clinical applications. Although affinity maturation is a promising antibody engineering technology used to improve the biological activity of antagonistic antibodies,^1^ it is unlikely to be applicable to other types of antibodies, such as agonistic antibodies,^11^ catalytic antibodies,^12^ or our FVIII-mimetic bispecific antibody, in which having a higher binding affinity to the antigen does not necessarily result in higher biological activity.^7^ In the case of our bispecific antibody, the antibody needs to bind to both FIXa and FX with adequate affinity to promote the interaction between the factors. Then, after FX activation by FIXa, FXa is required to be rapidly released from the antibody to proceed to the subsequent coagulation reaction, and to enable the antibody to turn over. Therefore, the binding kinetics (*k*_on_, *k*_off_ and *K*_D_) to both FIXa and FX should be optimized to maximize the FVIII-mimetic activity. More importantly, since distance and angle between the two factors are essential, the tertiary structure of the bispecific antibody, mainly that of its two Fab arms, can strongly influence the FVIII-mimetic activity. Thus, antibody modifications in the non–antigen-contacting region, which could potentially affect the tertiary structure without affecting the antigen-binding affinity, may also improve the FVIII-mimetic activity.

Hence, in this study, we investigated the effects of modifications to the non–antigen-contacting regions on the FVIII-mimetic activity of lead bispecific antibodies. We first examined multiple symmetric and asymmetric combinations of heavy-chain human IgG subclasses (IgG_1_, IgG_2_, IgG_4_), and engineered the non–antigen-contacting region, including the inter-chain disulfide bonds, upper hinge region, elbow hinge region,^13,14^ and Fc glycosylation at Asn297, any of which could potentially affect the tertiary structure or flexibility of the bispecific IgG antibody. Most of the modifications tested in this report significantly affected the FVIII-mimetic activity, and the mutations altering the disulfide bonds between the heavy and light chains and those between the two heavy chains had the largest effect.

## Results

### Effect of IgG subclass on FVIII-mimetic activity

**Figure 1.**
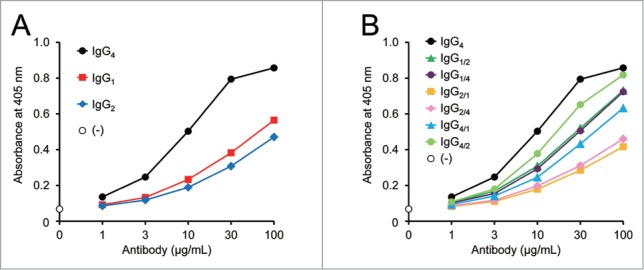
Effect of IgG subclass on FVIII-mimetic activity. Effect of each antibody on FIXa-catalyzed FX activation in the presence of FIX, FIXa, FX, and synthetic phospholipid is shown. The X-axes indicate the concentration of antibody in the FX activation reaction. The Y-axes indicate the absorbance at 405 nm after the addition of chromogenic substrate S-2222, which represents the generation of FXa. (**A**) The parent human IgG_4_ antibody with Ser228Pro (black circles) shows the antibody concentration–dependent FXa generation, described as the FVIII-mimetic activity. The FVIII-mimetic activity of the human IgG_1_ variant (red squares), and the human IgG_2_ variant with the IgG_1_-like disulfide bond pattern (blue diamonds) is shown. (**B**) The FVIII-mimetic activity of the parent IgG_4_ antibody (black circles) and the different subclass combinations: IgG_1/2_ (green triangles), IgG_1/4_ (purple circles), IgG_2/1_ (yellow squares), IgG_2/4_ (pink diamonds), IgG_4/1_ (light blue triangles), and IgG_4/2_ (light green circles) is shown. The number before the slash indicates the subclass of the Q chain, and the number after the slash indicates the subclass of the J chain, e.g. the Q and J chain subclasses of IgG_1/2_ are IgG_1_ and IgG_2_, respectively.

In order to investigate the effects of the tertiary structure of this asymmetric bispecific IgG antibody on the FVIII-mimetic activity, we evaluated the influence of IgG subclasses, human IgG_1_, IgG_2_ and IgG_4_. Instead of using wild-type human IgG_4_ and IgG_2_, we used the human IgG_4_ variant with a hinge stabilizing mutation (Ser228Pro),^15^ and the human IgG_2_ variant with two cysteine to serine mutations in the C_H_1 and hinge regions (Cys131Ser and Cys219Ser mutations result in the SCVE upper hinge sequence in one-letter abbreviation instead of the wild-type CCVE sequence), to minimize the disulfide bond-related heterogeneity of wild-type human IgG_2_.^16^ Knobs-into-holes mutations^17^ were introduced into the C_H_3 region of all the IgG subclasses used in this study to promote the heterodimerization of the Q and J chains. The FVIII-mimetic activity of bispecific antibodies was evaluated in an enzymatic assay by measuring the FIXa-catalyzed FX activation enhanced either by FVIII or by FVIII-mimetic cofactors. The parent human IgG_4_ antibody enhanced FX activation dose-dependently in this enzymatic assay, demonstrating its FVIII-mimetic activity (**Fig. 1A**). The IgG subclasses affected the FVIII-mimetic activity, suggesting that some structural differences among the IgG subclasses are important for the activity of this antibody.

Moreover, asymmetric IgG subclass combinations, with a different IgG subclass in the Q and J chains, were examined. These asymmetric variants showed a variety of FVIII-mimetic activity, and interestingly, the IgG_4/2_ variant with the IgG_4_ Q chain and IgG_2_ J chain showed significantly higher activity than the IgG_2/4_ variant with the IgG_2_ Q chain and IgG_4_ J chain (**Fig. 1B**). This result demonstrates that altering the sequence affects the FVIII-mimetic activity asymmetrically, which is reasonable when the mode of action of this bispecific antibody is considered.

### Effect of disulfide bond formation between heavy and light chains on FVIII-mimetic activity

**Figure 2.**
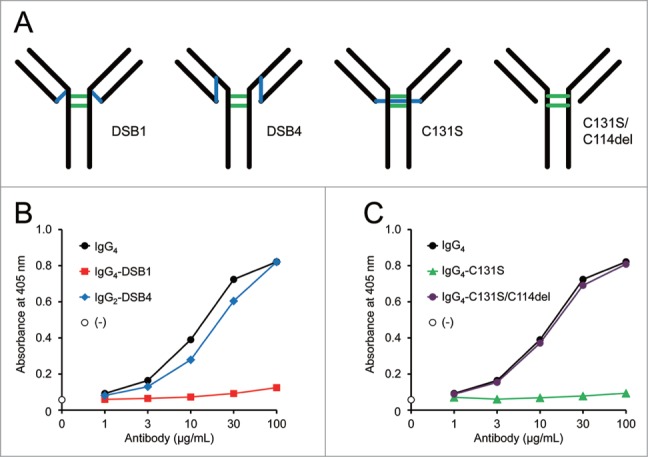
Effect of disulfide bonds between heavy and light chains on FVIII-mimetic activity. (**A**) Schematic structures of the IgG_1_-like disulfide bond formation (DSB1), IgG_4_-like disulfide bond formation (DSB4), the IgG_4_-C131S variant, and IgG_4_-C131S/C114del variant are shown. The blue lines indicate the disulfide bonds between the heavy and light chains or between the two light chains. The green lines indicate disulfide bonds between the two heavy chains. (**B**) The FVIII-mimetic activity of the parent IgG_4_ antibody (black circles), the IgG_4_-DSB1 variant (red squares), the IgG_2_-DSB4 variant (blue diamonds) is shown. (**C**) The FVIII-mimetic activity of the parent IgG_4_ antibody (black circles), the IgG_4_-C131S variant (green triangles), and the IgG_4_-C131S/C114del variant (purple circles) is shown.

One of the structural differences between the IgG subclasses is the disulfide bond pattern between the heavy and light chains. The human IgG_1_ and IgG_2_ variants used in this study form disulfide bonds between the heavy chain through Cys220 in the hinge region (upper hinge region with SCDK sequence for the IgG_1_ variant, and SCVE sequence for the IgG_2_ variant) and the light chain Cys114 at the C-terminal of the C_L_ (DSB1, **Fig. 2A**). Human IgG_4_ antibodies form disulfide bonds between the heavy chain through Cys131 in the C_H_1 region and the light chain Cys114 at the C-terminal of the C_L_ (DSB4, **Fig. 2A**).^18^ To investigate the effects of the disulfide bond pattern on the FVIII-mimetic activity, the IgG_4_ and IgG_2_ variants with different disulfide bond patterns between the heavy and light chains were examined. The IgG_4_ variant, IgG_4_-DSB1, which has Cys131Ser and Gly220Cys mutations that form an IgG_1_-like disulfide bond pattern^19^ with the linkage between the heavy chain Cys220 in the hinge region (upper hinge with YCPP sequence) and the light chain Cys114 (DSB1, **Fig. 2A**), showed surprisingly much less FVIII-mimetic activity than the parent IgG_4_ antibody (**Fig. 2B**). In addition, the IgG_2_ variant, IgG_2_-DSB4, which has cysteine at the 131^st^ position and no cysteine in the hinge region (upper hinge with SSVE sequence) to form an IgG_4_-like disulfide bond pattern with the linkage between the heavy chain Cys131 and the light chain Cys114 (DSB4, **Fig. 2A**), showed FVIII-mimetic activity similar to that of the parent IgG_4_ antibody (**Fig. 2B**). These results demonstrate that the disulfide bond pattern between the heavy and light chains significantly affects the FVIII-mimetic activity of the bispecific antibody, and that the IgG_4_-like disulfide bond pattern seems to be important for FVIII-mimetic activity.

To further elucidate the importance of the IgG_4_-like disulfide bond pattern, other IgG_4_ variants with different disulfide bond patterns were examined. The cysteine residues in the C_H_1 and C_L_ regions were substituted or deleted. The heavy chain Cys131Ser variant, IgG_4_-C131S, was expected to have an unusual disulfide bond between Cys114 of the two light chains as previously reported,^20^ and the heavy chain Cys131Ser and light chain C-terminal cysteine deleted (C114del) double mutant, IgG_4_-C131S/C114del, forms no disulfide bond between the heavy and light chains (**Fig. 2A**). Surprisingly, IgG_4_-C131S/C114del exhibited activity comparable with the parent IgG_4_ antibody, whereas IgG_4_-C131S displayed almost no FVIII-mimetic activity (**Fig. 2C**). These results suggest that the IgG_4_ structure, determined by the disulfide bonds formed between the heavy chain Cys131 and light chain Cys114, is critically important for the activity of this bispecific IgG antibody.

### Effect of disulfide bond formation between two heavy chains on FVIII-mimetic activity

**Figure 3.**
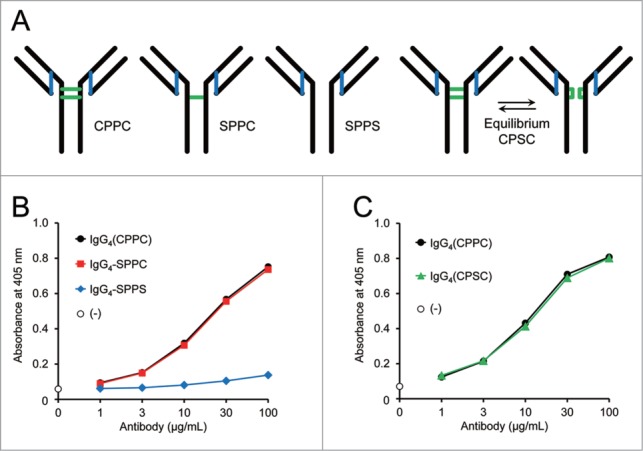
Effect of disulfide bonds between two heavy chains on FVIII-mimetic activity. (**A**) Schematic structures of the parent IgG_4_(CPPC) variant, the IgG_4_-SPPC variant, the IgG_4_-SPPS variant, and wild-type IgG_4_(CPSC) are shown. The blue lines indicate the disulfide bonds between the heavy and light chains. The green lines indicate the inter–and intra-heavy chain disulfide bonds. The wild-type IgG_4_(CPSC) is known to have equilibrium between the inter–and intra-heavy chain disulfide bonds. (**B**) The FVIII-mimetic activity of the parent IgG_4_(CPPC) antibody (black circles), IgG_4_-SPPC variant (red squares), and IgG_4_-SPPS variant (blue diamonds) is shown. (**C**) The FVIII-mimetic activity of the parent IgG_4_(CPPC) antibody (black circles) and the wild-type IgG_4_(CPSC) antibody (green triangles) is shown.

Next, the effect on FVIII-mimetic activity of the disulfide bonds between the two heavy chains at the hinge region of the bispecific IgG antibody was evaluated. The mutations Cys226Ser and Cys229Ser in the hinge region of the parent human IgG_4_ variant (hinge with CPPC sequence) were examined. The IgG_4_-SPPC variant with the Cys226Ser substitution conceivably has one disulfide bond between the Q and J chains, and the IgG_4_-SPPS variant with no cysteine residue in the hinge region does not have any disulfide bond between the two heavy chains (**Fig. 3A**). While the IgG_4_-SPPC variant maintained the FVIII-mimetic activity, the IgG_4_-SPPS variant showed significantly reduced activity (**Fig. 3B**). These results indicate that the presence of at least one disulfide bond between the two heavy chains seems to be necessary for FVIII-mimetic activity, presumably by reducing the flexibility or mobility of the two Fab arms against FIXa and FX. This result motivated us to test the effect of the wild-type human IgG_4_ hinge sequence (CPSC with serine at the 228^th^ position) on FVIII-mimetic activity, because the wild-type human IgG_4_ hinge is reported to have equilibrium between the inter-heavy chain disulfide bonds and the intra-heavy chain disulfide bonds with no inter-heavy chain disulfide bond (**Fig. 3A**).^18,20^ However, the FVIII-mimetic activity of wild-type human IgG_4_ with the CPSC hinge sequence was found to be comparable to that of human IgG_4_ variant with the CPPC hinge sequence (**Fig. 3C**).

### Effect of hinge flexibility on FVIII-mimetic activity

**Figure 4.**
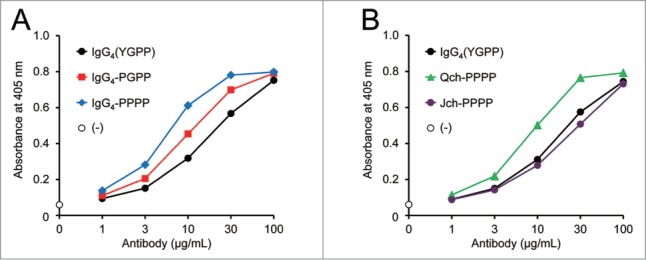
Effect of upper hinge sequence on FVIII-mimetic activity. (**A**) The FVIII-mimetic activity of the parent IgG_4_ antibody with the YGPP sequence (black circles), the IgG_4_-PGPP variant (red squares), and the IgG_4_-PPPP variant (blue diamonds) is shown. (**B**) The FVIII-mimetic activity of the parent IgG_4_(YGPP) antibody (black circles), the asymmetric upper hinge variants: Qch-PPPP (green triangles), and Jch-PPPP (purple circles), is shown.

A significantly reduced level of FVIII-mimetic activity by the IgG_4_-SPPS variant suggested that hinge flexibility was also likely to affect the FVIII-mimetic activity of the bispecific antibody, and that reduced flexibility of the two Fab arms of the bispecific antibody seems to be important for a high level of activity. Hence, proline residues, which would conceivably reduce the flexibility of the hinge structure, were introduced in the upper hinge region. The FVIII-mimetic activity of the original human IgG_4_ variant with the upper hinge sequence of YGPP and the two proline-introduced variants, IgG_4_-PGPP (with the Tyr219Pro mutation) and IgG_4_-PPPP (with the Tyr219Pro and Gly220Pro mutations), was examined. Introducing one proline residue (the IgG_4_-PGPP variant) increased the FVIII-mimetic activity, and introducing two proline residues (the IgG_4_-PPPP variant) further increased the activity (**Fig. 4A**). Because the asymmetric combination of different IgG subclasses had a significant effect on the activity, these proline mutations were introduced to each heavy chain. Interestingly, the PPPP upper hinge sequence was found to enhance FVIII-mimetic activity only when applied to the Q chain, but not when applied to the J chain (**Fig. 4B**).

### Effect of mutations in elbow hinge region on FVIII-mimetic activity

**Figure 5.**
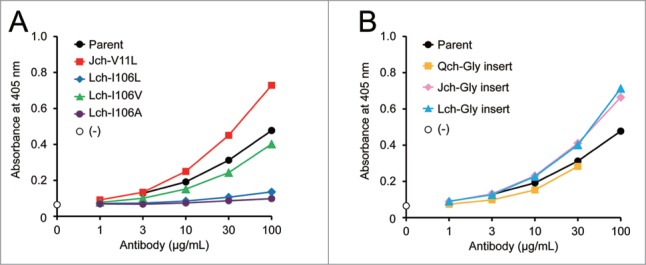
Effect of mutations in elbow hinge on FVIII-mimetic activity. (**A**) The FVIII-mimetic activity of the parent IgG_4_ antibody (black circles), Jch-V11L variant (red squares), Lch-I106L variant (blue diamonds), Lch-I106V variant (green triangles), and Lch-I106A variant (purple circles) is shown. (**B**) The FVIII-mimetic activity of the parent IgG_4_ antibody (black circles) and the glycine-inserted variants: Qch-Gly insert (yellow squares), Jch-Gly insert (pink diamonds), and Lch-Gly insert (light blue triangles), is shown.

The elbow angle or elbow hinge region is reported to influence the mobility of the Fv domain,^13,14^ which in turn could affect the FVIII-mimetic activity. Hence, we introduced various mutations to the elbow hinge region of the parent IgG_4_ antibody and found that the Val11Leu mutation in the J chain increased its activity, while the substitution of Ile106 to leucine and alanine, but not to valine, in the L chain significantly decreased its activity (**Fig. 5A**). Furthermore, we investigated the effects of inserting glycine residues in the boundary of the V_H_ and C_H_1 domains in the Q and J chains, and the boundary of the V_L_ and C_L_ domains in the L chain, which could also affect the Fv mobility of IgG antibodies. The insertion of glycine in the J and L chains significantly enhanced the FVIII-mimetic activity, while that in the Q chain slightly decreased the activity (**Fig. 5B**).

### Effect of Fc glycan on FVIII-mimetic activity

**Figure 6.**
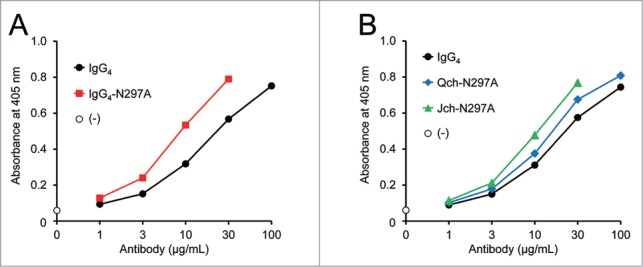
Effect of Fc glycosylation on FVIII-mimetic activity. (**A**) The FVIII-mimetic activity of the parent IgG_4_ antibody (black circles) and the deglycosylated N297A IgG_4_ variant (red squares) is shown. (**B**) The FVIII-mimetic activity of the parent IgG_4_ antibody (black circles) and the asymmetric deglycosylated Fc variants: Qch-N297A (blue diamonds), Jch-N297A (green triangles), is shown.

While it is well known that Fc glycan at Asn297 is critical for FcγR binding, Fc glycan also affects the tertiary structure of the Fc portion of IgG antibodies.^21^ The Asn297Ala mutation, which removes the N-glycosylation site in the C_H_2 domain, was examined to investigate the effect of the Fc portion structure on FVIII-mimetic activity. Surprisingly, Fc glycan was found to significantly affect the FVIII-mimetic activity, and removing the glycan enhanced the activity (**Fig. 6A**). The effect of introducing the Asn297Ala mutation to each heavy chain was also examined. The Asn297Ala mutations in the C_H_2 regions of the Q chain and J chain enhanced the activity (**Fig. 6B**). These results suggest that the Fc glycan indirectly affects the distance and angle between the two Fab arms of this bispecific antibody by altering the tertiary structure of the Fc portion.

## Discussion

The results of this study demonstrate that IgG subclass, inter-chain disulfide bonds, and mutations in the upper hinge region, elbow hinge region, and N-glycosylation site in the C_H_2 domain—areas which are unlikely to come into contact with the antigens directly—significantly affected the FVIII-mimetic activity. The structure of the constant region of a human IgG subclass is known to influence the flexibility of the two Fab arms and its binding characteristics to membrane-bound antigens.^5,22^ Our bispecific antibody brings the FIXa and FX into spatial proximity, and these two factors are bound to the phospholipid,^23^ making them similar to membrane-bound antigens. Thus, we expected that the FVIII-mimetic activity of our bispecific antibody would be strongly affected by the IgG subclass. We tested human IgG_1_, IgG_2_, and IgG_4_ subclasses and their asymmetric combinations, and found that IgG subclasses affect the activity asymmetrically. Although it is difficult to find a tendency that can be used to predict the factors affecting the activity only from this result, the symmetric IgG_4_ was found to be the most potent with respect to our bispecific antibody. This may be reasonable because we identified the lead bispecific antibody from a screening with symmetric IgG_4_, so the epitopes of our bispecific antibody for FIXa and FX were biased toward those that fit the tertiary structure of IgG_4_. Therefore, this suggests that the IgG subclass used for screening the lead antibody should be selected by considering the subclass of IgG that will be used for clinical development. Since mouse and rat IgGs have different structures from that of human IgG, screening this kind of functional antibody directly from hybridoma-derived antibodies should be avoided. Rather, as in the screening of the FVIII‑mimetic bispecific antibodies that were derived from mouse, rat, and rabbit, chimeric human IgG with an appropriate subclass should be used for functional screenings.

Almost complete loss of the FVIII-mimetic activity by the human IgG_4_ variant with an IgG_1_-like disulfide bond pattern (IgG_4_-DSB1) indicates that the structure determined by the IgG_1_-like disulfide bond pattern strongly inhibits FVIII-mimetic activity. Since the heavy and light chains interact non–covalently even in the absence of disulfide bonds between them, the tertiary structure of Fab seems not to be affected strongly by the presence of a disulfide bond between either the heavy chain Cys131 or Cys220 and the light chain C-terminal of Cys114. Therefore, it is likely that using Cys220 in the upper hinge region for a disulfide bond may affect the flexibility of the hinge region, the angle and distance of the two Fab arms, or the tertiary structure of whole IgG and, thereby, inhibit the FVIII-mimetic activity. To the best of our knowledge, this is the first report to show that the disulfide bond pattern between the heavy and light chains significantly affects the biological activity of antibodies. These findings also help explain the variation in the activity of the different IgG subclasses.

The disulfide bonds within the two heavy chains and the two light chains also affected the activity. The almost complete loss of activity of the IgG_4_-SPPS variant, in which the lack of disulfide bonds between the two heavy chains makes the two Fab arms more likely to be flexible, suggests that too flexible a structure of the bispecific antibody is not suitable to place FIXa and FX into spatial proximity efficiently. On the other hand, the IgG_4_-C131S variant, in which the presence of the non–natural disulfide bond linkage between the two light chains makes the two Fab arms more likely to be fixed, also showed an almost complete loss of activity. These results suggest that an optimal flexibility of the two Fab arms is important for the activity.

Wild-type human IgG_4_ antibody with the CPSC hinge sequence is unique in existing as a “half molecule,” composed of one heavy chain and one light chain that lack the inter-heavy chain disulfide bond, which can combine with one another in a process known as “Fab-arm exchange (FAE),” resulting in bispecific antibodies that are functionally monovalent in plasma.^24-26^ Ser228Pro was introduced into the hinge region of our bispecific IgG_4_ antibody in order to prevent FAE with endogenous IgG_4_ in vivo, because our bispecific antibody mimicking the cofactor function of FVIII is no longer effective if FAE occurs in vivo. The Ser228Pro mutation with the CPPC hinge sequence is known to restrict the torsional freedom equilibrium between inter–and intra-heavy chain cysteine residues.^20,24,26^ Compared to the stabilized CPPC hinge sequence, the CPSC hinge sequence may give more flexibility to the two Fab arms by enabling IgG_4_ antibody to form equilibrium between the inter–and intra-heavy chain disulfide bonds, which can result in a more flexible structure, like the IgG_4_-SPPS variant that lacks the inter-heavy chain disulfide bonds. Therefore, we tested the effect of the Ser228Pro mutation on the FVIII-mimetic activity. No distinct difference in the activity between the CPSC and CPPC IgG_4_ antibodies was observed. Because IgG_4_-SPPS permanently lacks the inter-chain disulfide bonds, it has much more flexible movement of the two Fab arms, which is likely to reduce the FVIII-mimetic activity significantly. On the other hand, conceivably because the conversion between the inter–and intra-chain disulfide bonds in IgG_4_-CPSC is too rapid to increase the flexibility of the two Fab arms, the activity of IgG_4_-CPSC was comparable to IgG_4_-CPPC. It has been reported that the lower hinge mobility and compact form of IgG_4_, which seems to affect the binding to FcγRs and C1q, is possibly an outcome of the short and constrained hinge structure of IgG_4_.^26^ This unique structure of IgG_4_ may be significant for the FVIII-mimetic activity of our bispecific antibody, and the result suggests that the Ser228Pro mutation may not change the tertiary structure of IgG_4_ dramatically.

The introduction of proline residues in the upper hinge region enhanced the FVIII-mimetic activity. These introduced proline residues may provide a more rigid upper hinge structure or a different Fab arm angle in the bispecific IgG antibody. Kai et al. reported that the agonistic activity of an anti-thrombopoietin receptor antibody can be improved by using the IgG_3_ hinge sequence with a more flexible upper hinge structure.^4^ Engineering the upper hinge region could be a general approach to modulating the agonist-like activity of antibodies.

The elbow hinge region is located at the interface of Fv and C_H_1/C_L_ and has been known to affect the mobility and angle of the Fv domain.^13,14^ During the humanization process of our lead antibody, we found that humanizing the mouse-derived J chain decreased but that of the mouse/rat-derived L chain increased the FVIII-mimetic activity, which, in turn, the activity of whole bispecific antibody was not affected by the humanization as we previously reported.^7^ We noticed that the 11th position of the J chain and the 106th position of the L chain of the mouse/rat antibody were leucine, which the humanization process changed to valine and isoleucine, respectively. These findings motivated us to substitute these positions in the humanized J and L chains. The Val11Leu mutation in the J chain increased the FVIII-mimetic activity, while the Ile106Leu or Ala mutation, but not the Ile106Val mutation, in the L chain significantly decreased the activity. On the other hand, the humanization process did not change the residue at the 11th position in the rat-derived Q chain (both rat and humanized Q chains had leucine at the 11th position) and thus did not affect the FVIII-mimetic activity; therefore, we did not study the effect of the 11th position in the Q chain. Both the 11th position in the heavy chain and the 106th position in the light chain, which form hydrophobic interactions, are buried in the interface of the variable and constant regions, and generally do not directly interact with the antigens or affect the conformation of the complementarity-determining regions (CDRs). Because the residues were mutated to become hydrophobic residues, the variants would not significantly disrupt the hydrophobic interaction. Nevertheless, a significant effect on the FVIII-mimetic activity was observed, possibly due to a slight change in the mobility and angle of the Fv domains. Previously, Mossner et al. reported that the same 11th position of the heavy chain in the anti-CD20 antibody obinutuzumab was found to be the key residue for its apoptosis-inducing activity, when Val11Leu mutation significantly reduced the cell death induction activity, but did not affect its binding affinity to the antigen.^27^ These results suggest that the elbow hinge region of the antibody, including the 11th position of the heavy chain, significantly contributes to the biological activity of antibodies that are not simple antagonistic antibodies.

Stanfield et al. reported that the elbow angles are influenced by the light chain subclass.^28^ λ light chain gives more flexibility to the Fv region than κ light chain because of an extra residue in the boundary region between the variable and constant regions. To mimic the increased flexibility of λ light chain, a glycine residue was inserted in the boundary region of the κ light chain of our bispecific antibody. The FVIII-mimetic activity was enhanced by inserting glycine in the L chain, presumably because this increased the flexibility or modified the angle of the Fv region. However, when we replaced the whole of framework 4 and the constant region sequence with those of a λ light chain, the activity was significantly reduced (data not shown). This suggests that a glycine insertion in the κ light chain and the extra residue of the λ light chain each modifies the flexibility or angle of the Fv domains and affects the FVIII-mimetic activity differently. Inserting glycine in the boundary of the V_H_ and C_H_1 domains in the two heavy chains was also tested. Whereas its insertion into the J chain enhanced the activity, its insertion into the Q chain slightly reduced the activity. These results suggest that flexibility of the elbow angles of the Fv significantly affects the activity of our bispecific antibody, and the glycine insertion can be a novel approach to modulating agonist-like activity of antibodies.

The effect of Fc glycosylation on the biological activity of antibodies has mainly been studied with regard to FcγR binding.^2,3,29,30^ Because a deglycosylated antibody shows diminished FcγR binding, it has been widely used in antibody therapeutics where FcγR-mediated effector function is undesirable. Surprisingly, we found that using the Asn297Ala mutation to remove the N-glycosylation sites increased the FVIII-mimetic activity, even though this activity is unrelated to FcγR binding. It has been reported that removing the Asn297 glycan results in a more “closed” Fc conformation, bringing the two C_H_2 domains closer in proximity compared to fully glycosylated Fc with an “open” conformation.^21^ One explanation for the enhanced FVIII-mimetic activity by Fc deglycosylation is that the Fc deglycosylation indirectly changes the hinge flexibility by taking “closed” Fc conformation. Another explanation could be that the reported interaction between the C_H_1 and C_H_2 domains of IgG_4_^26^ is modified by a conformational change of the C_H_2 domains, which resulted in changing the distance and angle between the two Fab arms.

In this study, we have also generated several asymmetric variants of the heavy chain constant region. Considering the mode of action of our bispecific antibody, asymmetric engineering may have a different effect from symmetric engineering. Human IgG subclass asymmetric variants with a different IgG subclass for the two heavy chains showed a variety of FVIII-mimetic activity. Although the higher activity of the IgG_4/2_ variant compared to the IgG_2/4_ variant suggests the importance of an IgG_4_-like structure in the Q chain for the activity, this was not the case for IgG_4/1_ and IgG_1/4_. In addition, while the enhanced activity by proline mutations in the hinge region was effective only in the Q chain, the removal of the Fc glycan in either the Q or J chain enhanced the activity. Recently, Mimoto et al. reported that asymmetric engineering of the C_H_2 domain could be applied to optimize Fc-FcγR interaction.^31^ This strategy is effective since Fc interacts with FcγRs in an asymmetric manner^32^ and indeed, our bispecific antibody also interacts with FIXa and FX in an asymmetric manner. Although we have not obtained a concrete understanding of the effects of asymmetric engineering, our results demonstrate that asymmetric engineering can be applied to enhance the activity of our bispecific antibody. We speculate that asymmetric engineering can also be applied to a similar type of antibody that interacts with the target(s) in an asymmetric manner.

We demonstrated that the engineering of the non–antigen-contacting region significantly affected the FVIII-mimetic activity. It is difficult to further discuss how these mutations alter the tertiary structure of the bispecific antibody and affect the FVIII-mimetic activity because an X-ray structure of whole human IgG_4_ is not publicly available to date. Although we tried to acquire the binding kinetics of our variants to FIXa and FX, we were not able to obtain meaningful kinetic parameters because of the very low binding affinity of our bispecific antibodies to both FIXa and FX, as previously discussed.^7^ We assume that most of the mutations would not be likely to affect the binding affinity because they are introduced in the non–antigen-contacting regions. Nevertheless, we cannot disprove the possibility that some of them may have affected the binding affinity. Hence, this study suggests that non–antigen-contacting regions have a significant effect on the FVIII-mimetic activity, by altering the tertiary structure and the flexibility of the antibody, and potentially by indirectly altering the binding affinity to FIXa or FX (in other words, the change in tertiary structure and flexibility may indirectly affect the binding affinity).

Since we identified several mutations that enhanced the activity, some combinations of these mutations were examined. An additive effect of the combination of the Val11Leu mutation and the glycine insertion in the boundary of the V_H_ and C_H_1 domains in the J chain was observed as shown in **Figure S1**. Introducing proline residues and the Fc deglycosylation mutation to an optimized variant that already had multiple mutations in the CDRs, elbow hinge regions, and the boundary of the variable and constant regions also increased the FVIII-mimetic activity additively as shown in **Figure S2**. This suggests that types of engineering in the non–antigen-contacting regions can be combined to generate bispecific antibodies with improved FVIII-mimetic activity.

The FVIII-mimetic activity of our bispecific antibodies measured by an enzymatic assay shows a good correlation with a thrombin generation assay using commercial FVIII-deficient human plasma that was derived from a severe hemophilia A patient,^6,7^ and the activity shown by that assay also correlates with the in vivo efficacy in a cynomolgus monkey acquired hemophilia model.^6,8^ Therefore, we expect that the effects of the non–antigen-contacting region on the in vivo efficacy would be in line with the FVIII-mimetic activity as measured by the enzymatic assay described in this paper.

Mutations in the non–antigen-contacting region described in this study may also affect pharmacokinetics, stability, and productivity of the bispecific antibody. Although they were not tested, we assume that these mutations would not affect the pharmacokinetics because they are not located in the neonatal Fc receptor (FcRn) binding region. The stability and production yield were actually affected by some of the mutations. A reduction in thermal stability of the Fab domain was observed when a glycine residue was inserted in the boundary of the variable and constant regions possibly because the interface of the Fv and C_H_1/C_L_ was destabilized (data not shown). The Asn297Ala mutation decreased the thermal stability of the C_H_2 domain and production yield (data not shown).

In summary, we have tested the effects of engineering the non–antigen-contacting region in an asymmetric anti-FIXa/FX bispecific IgG antibody and observed that the engineering significantly affected the FVIII-mimetic activity, presumably by modulating the tertiary structure of the IgG or by modifying the flexibility of the two Fab arms. We identified several variants with mutation(s) in the upper hinge region, elbow hinge region, and N-glycosylation sites that improved the FVIII-mimetic activity. However, the extent of improvement to the activity was much less than that achieved by optimizing the antigen-contacting region of the bispecific antibody, which we previously reported.^7^ Considering the potential risk of instability and immunogenicity, such activity-improving mutations in the constant region were not introduced in ACE910, which is currently being tested in a clinical trial. Nevertheless, our study clearly demonstrates the importance of the non–antigen-contacting regions for the activity of our bispecific IgG antibody. It is expected that these non–antigen-contacting regions can be engineered to improve the biological activity of IgG antibodies with functions similar to ACE910, such as placing two antigens into spatial proximity, retargeting effector cells to target cells, or co-ligating two identical or different antigens on the same cell.

## Materials and Methods

### Generation of anti-FIXa/FX bispecific antibody variants

An asymmetric humanized bispecific IgG_4_ antibody with the cofactor function of coagulation FVIII was generated as previously reported.^7^ The regions of the Q, J, and L chains that do not have contact with the antigen were engineered to investigate the effect of each sequence alteration on the FVIII-mimetic activity. A lead humanized bispecific IgG_4_ antibody hBS1^7^ was used to study the effect of mutations in the elbow hinge region, glycine insertion, and their combinations. hBS10, an optimized variant of hBS1, was used to study the effect of the IgG subclass and the wild-type IgG_4_ hinge sequence. hBS13, an optimized variant of hBS10, was used to study the effect of the disulfide bonds between the heavy and light chains or between the two heavy chains, the upper hinge sequence, and the Fc deglycosylation. hBS20, an optimized variant of hBS13, was used to study the effect of combining the proline introduction and the Fc deglycosylation.

### Expression and purification of bispecific antibodies

Each bispecific antibody used in this report consists of two different heavy chains and two identical common light chains. Knobs-into-holes mutations were used to promote heterodimerization of the two heavy chains.^17^ Bispecific antibody variants were expressed by HEK293 cells co-transfected with a mixture of three expression plasmids encoding the Q, J, and L chains. These antibodies were purified by using protein A.

### Evaluation of FVIII-mimetic activity of bispecific antibodies (enzymatic assay)

The ability of each antibody to enhance FIXa-catalyzed FX activation was evaluated in an enzymatic assay using purified human coagulation factors. The FXa generation reaction was performed in the presence of 0.1 nM human FIXa (Enzyme Research Laboratories), 1 IU/mL human FIX (Chemo-Sero-Therapeutic Research Institute), 140 nM human FX (Enzyme Research Laboratories), 62.5 μM synthetic phospholipid (10% phosphatidylserine, 60% phosphatidylcholine and 30% phosphatidylethanolamine; Avanti Polar Lipids) prepared as previously described,^33^ and antibodies at room temperature for 10 min in TBS containing 5 mM CaCl_2_, 1 mM MgCl_2_, and 0.1% (wt/vol) BSA (pH 7.6). The reaction was stopped by the addition of EDTA at appropriate time points. The activity of the generated FXa was determined by absorbance at 405 nm after the addition of chromogenic substrate S-2222 (Chromogenix). All data was collected in singlet.
