# Supplementary material for: Non–antigen-contacting region of an asymmetric bispecific antibody to factors IXa/X significantly affects factor VIII-mimetic activity
Source: MAbs. 2014 Dec 18;7(1):120–8. doi: 10.4161/19420862.2015.989028 (PMC4622617; doi:10.4161/19420862.2015.989028)
Supplement: Supplemental_Figure_2.pdf [file kmab-07-01-989028-s001.pdf]

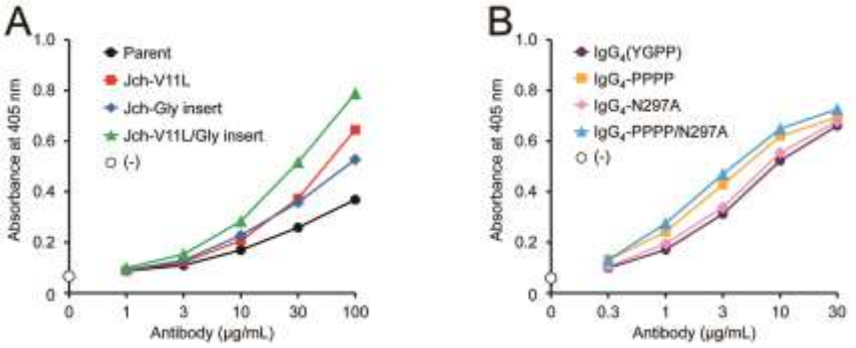

Supplemental Figure Effect of combination of mutations on FVIII-mimetic activity. (**A**) The FVIII-mimetic activity of the parent IgG_4 antibody (black circles) is compared with that of the J chain variant with the Val11Leu mutation (Jch-V11L, red squares), the variant with a glycine insertion in the boundary of the V_H and C_H1 domains in the J chain (Jch-Gly insert, blue diamonds) and their combination variant (Jch-V11L/Gly insert, green triangles). (**B**) The FVIII-mimetic activity of the parent IgG_4 antibody with the YGPP sequence (purple circles) is compared with that of the proline introduced variant (IgG_4-PPPP, yellow squares), the deglycosylated variant (IgG_4-N297A, pink diamonds) and their combination variant (IgG_4-PPPP/N297A, light blue triangles).
