# Supplementary figures and images for: Non–antigen-contacting region of an asymmetric bispecific antibody to factors IXa/X significantly affects factor VIII-mimetic activity
Source: MAbs. 2014 Dec 18;7(1):120–8. doi: 10.4161/19420862.2015.989028 (PMC4622617; doi:10.4161/19420862.2015.989028)

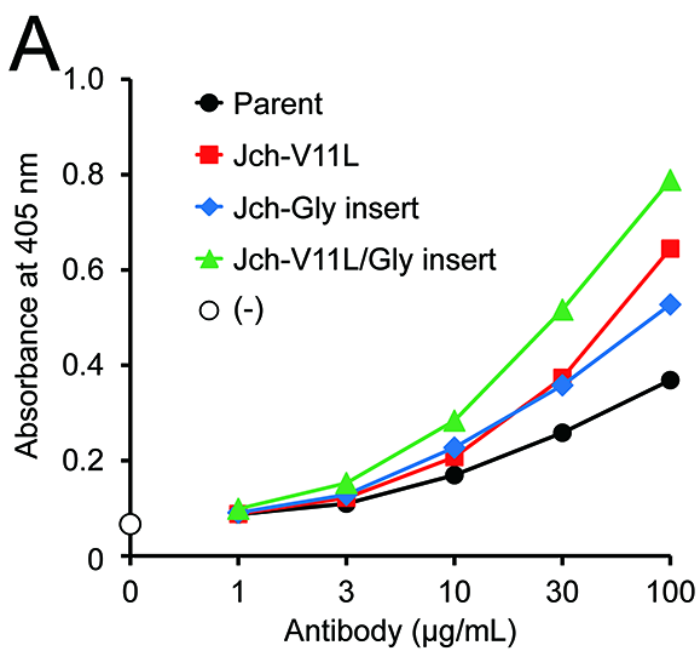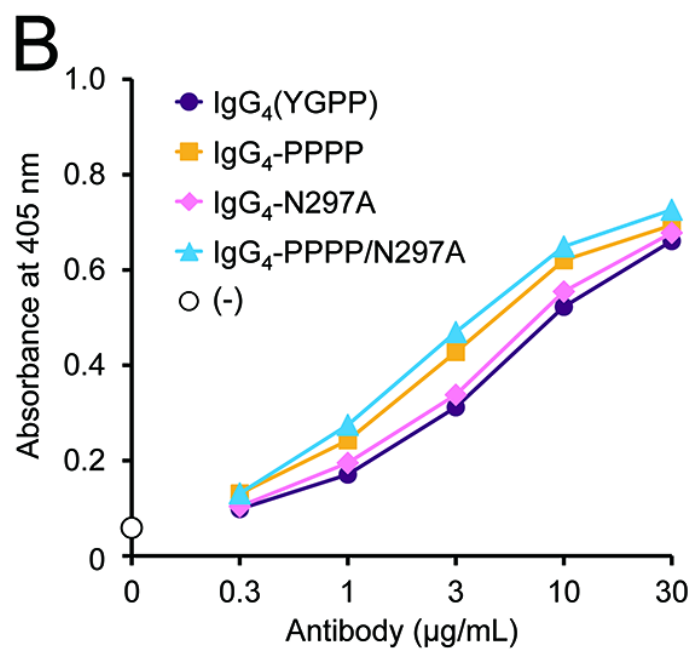

Supplement: Supplemental_Figure_1.pdf [file kmab-07-01-989028-s002.pdf]
